# Treatable Traits‐Oriented Management in Pre‐COPD With Severe Obstructive Sleep Apnea: A Case Report of Symptomatic and Spirometric Improvement After Integrated Therapy

**DOI:** 10.1002/rcr2.70710

**Published:** 2026-08-07

**Authors:** Norifumi Kudeken

**Affiliations:** ^1^ Kubagawa Medical Clinic Naha Okinawa Japan

**Keywords:** COPD Assessment Test, inhaled triple therapy, obstructive sleep apnea, pre‐COPD, treatable traits

## Abstract

Pre‐chronic obstructive pulmonary disease (pre‐COPD) is an early disease state characterised by respiratory symptoms and/or structural abnormalities without established airflow limitation. We report a woman in her 40s with cough, exertional dyspnoea, preserved spirometry, mild emphysematous change and severe obstructive sleep apnea (apnea‐hypopnea index, 88.1 events/h). Integrated management with inhaled fluticasone furoate/umeclidinium/vilanterol and continuous positive airway pressure was initiated. After 12 weeks, FEV_1_ increased from 2.27 to 2.73 L and FEV_1_/FVC from 82.5% to 86.9%, while the COPD Assessment Test score decreased from 12 to 3. The patient continued smoking and body weight remained essentially stable. Systemic inflammatory markers showed no clear overall improvement. Eosinophilic asthma was considered less likely because bronchodilator responsiveness was negative, blood eosinophils and total IgE were low, sputum eosinophils were negative, and sputum cytology showed neutrophilic inflammation. This case is hypothesis‐generating and suggests that selected pre‐COPD patients may benefit from multifactorial trait‐oriented management.

## Graphical abstract

This case illustrates a multifactorial treatable traits‐oriented approach in pre‐COPD complicated by severe obstructive sleep apnea. Inhaled triple therapy plus effective CPAP was associated with symptomatic and spirometric improvement over 12 weeks, although causality cannot be inferred from a single case.

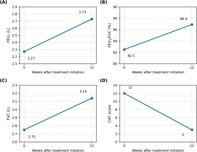

## 1 Introduction

Pre‐chronic obstructive pulmonary disease (pre‐COPD) is increasingly recognised as a clinical state characterised by respiratory symptoms and structural or functional abnormalities, such as airway disease or emphysema, in the absence of established airflow limitation on spirometry [1, 2]. This concept reflects evidence that tobacco‐exposed individuals with preserved FEV_1_/FVC ratios may already have clinically relevant respiratory impairment and may be at risk of progression. However, optimal management strategies remain uncertain.

Evidence supporting pharmacological intervention in symptomatic tobacco‐exposed individuals with preserved spirometry is limited. In the RETHINC trial, dual bronchodilator therapy did not significantly improve symptoms in such individuals [3]. These findings suggest that bronchodilation alone may be insufficient in some patients and that coexisting modifiable traits should be systematically identified.

Obstructive sleep apnea (OSA) may contribute to respiratory symptoms, sleep fragmentation, intermittent hypoxaemia and systemic inflammation [4]. The treatable traits framework emphasizes identification and targeted treatment of modifiable clinical traits rather than reliance on spirometric thresholds alone. We report a case of pre‐COPD complicated by severe OSA in which integrated management was associated with symptomatic and spirometric improvement while acknowledging alternative explanations and the limitations of a single case.

## 2 Case Report

A woman in her 40s presented with chronic cough and exertional dyspnoea. She was an office worker and had no history of occupational dust exposure. She had been exposed to household secondhand smoke during childhood and adolescence and started active smoking at 20 years of age, with a cumulative smoking exposure of 15 pack‐years. She remained a current smoker during the 12‐week follow‐up period. No structured smoking‐cessation counselling programme or smoking‐cessation pharmacotherapy was initiated during follow‐up. Body weight remained essentially stable. Relevant comorbidities included hypertension, treated at another clinic and Chiari malformation type I, which was being followed conservatively with headache medication. No additional oral corticosteroids, antibiotics, antiallergic agents, proton pump inhibitors, mucolytics, or other relevant medications were added during follow‐up.

**FIGURE 1:**
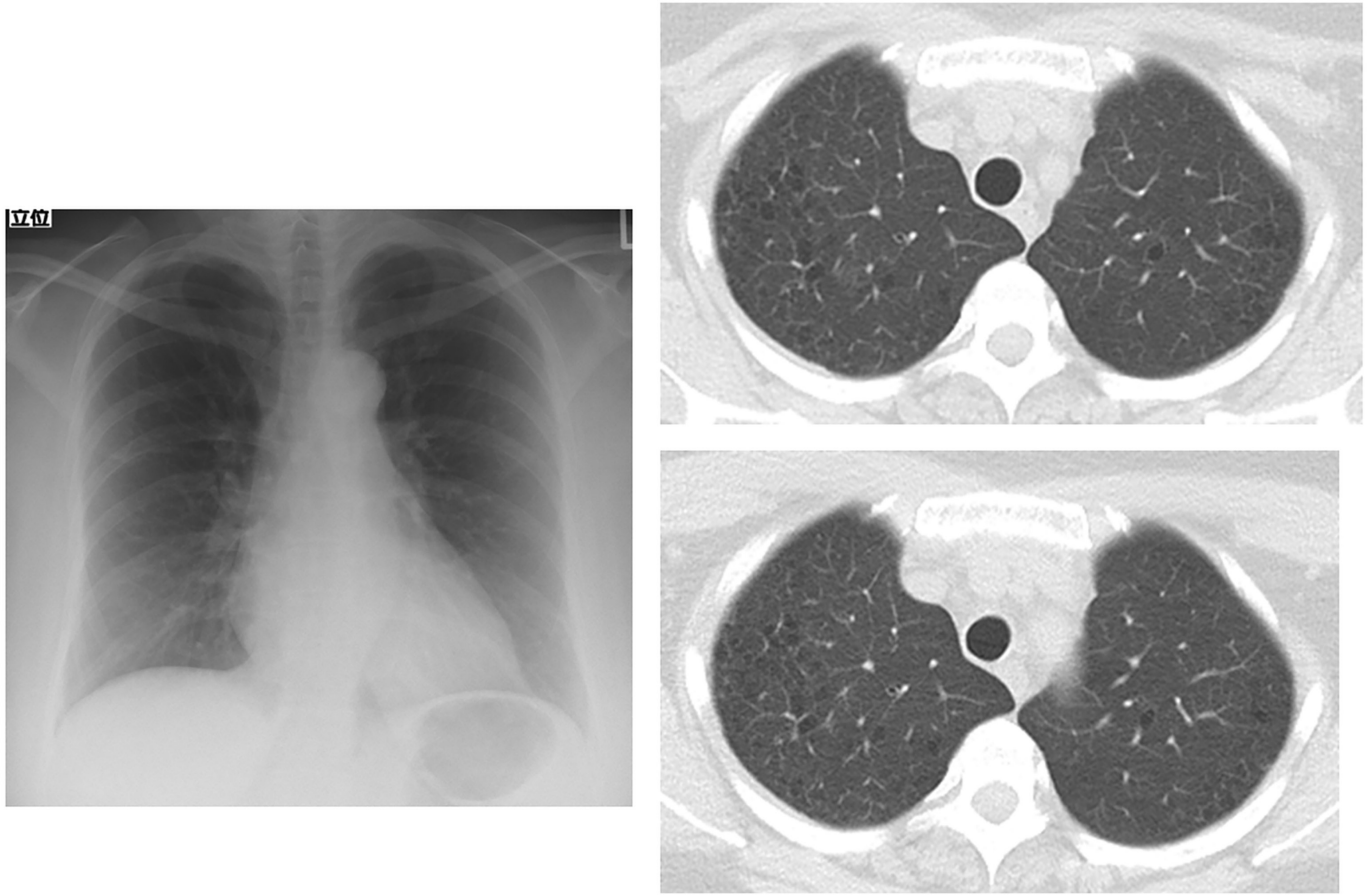
Baseline chest computed tomography showing mild centrilobular emphysematous change.

Post‐bronchodilator baseline spirometry showed preserved airflow without obstruction: FVC 2.75 L (91.6% predicted), FEV_1_ 2.27 L (88.3% predicted) and FEV_1_/FVC 82.5%. Chest computed tomography (Figure 1) showed mild emphysematous changes, consistent with pre‐COPD in the context of respiratory symptoms and smoking exposure. Polysomnography demonstrated severe OSA, with an apnea‐hypopnea index of 88.1 events/h, oxygen desaturation index of 80.2 events/h, minimum oxygen saturation of 49%, mean oxygen saturation of 89% and 178 min with oxygen saturation below 90%.

Respiratory phenotyping did not support prominent eosinophilic asthma. No wheeze was heard on chest auscultation at any visit during the clinical course. Pre‐treatment bronchodilator responsiveness testing showed an increase in FEV_1_ from 2.08 to 2.27 L (+190 mL, +9.1%), which did not meet the conventional criteria for a significant bronchodilator response. Peripheral blood eosinophil counts were low (96 cells/μl), total IgE was low (11 IU/mL), sputum eosinophils were negative and sputum cytology showed neutrophilic airway inflammation. Fractional exhaled nitric oxide (FeNO) was not measured.

Integrated therapy was initiated with inhaled fluticasone furoate/umeclidinium/vilanterol 100/62.5/25 μg once daily in the morning (Trelegy 100 Ellipta) and continuous positive airway pressure (CPAP) for severe OSA. Adherence to inhaled therapy was good by patient report, although inhaler technique was not formally assessed. CPAP adherence was excellent, with average use of 7 h 50 min/night, use on 100% of days, use for at least 4 h on 100% of days and residual AHI of 3 events/h. No adverse events were reported.

**FIGURE 2:**
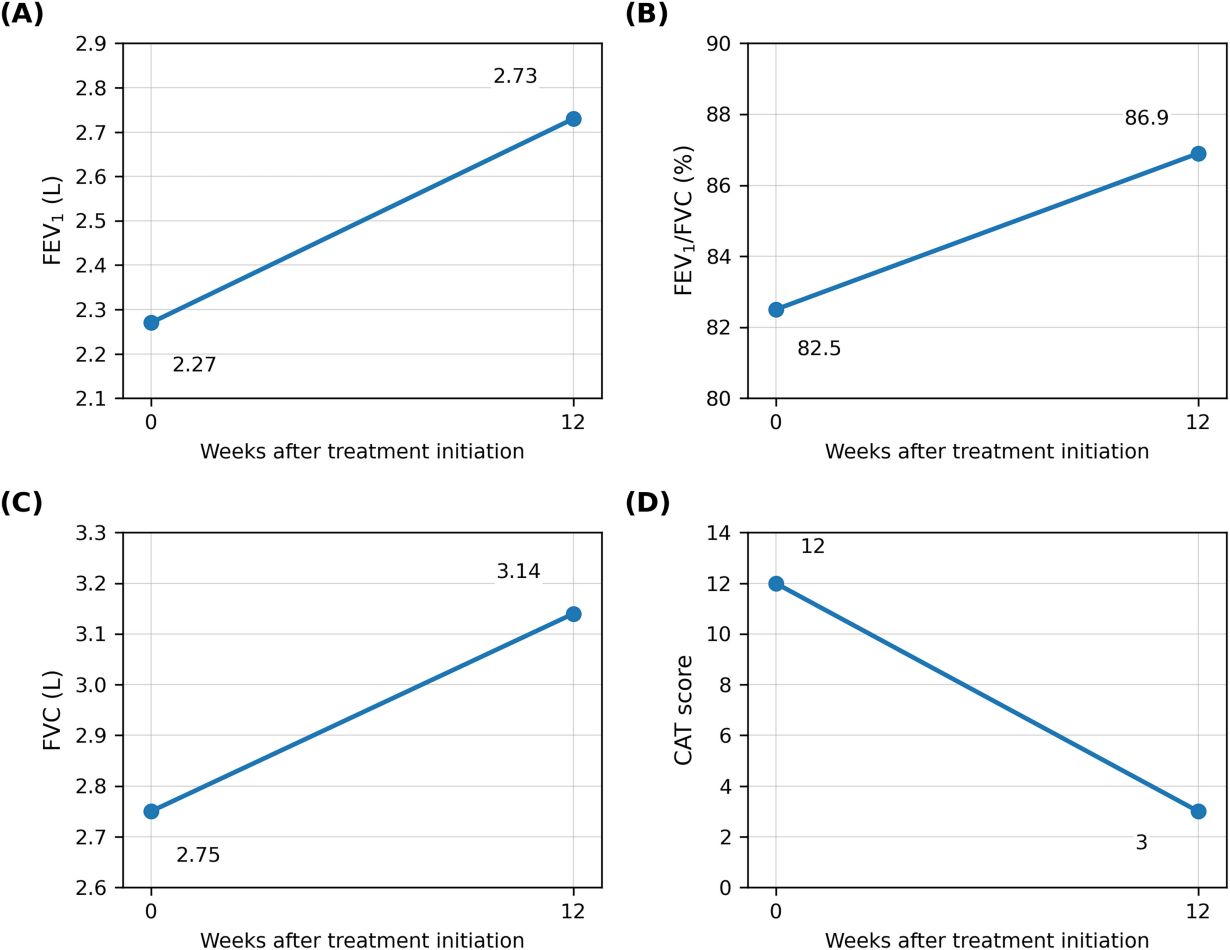
Changes in spirometry and COPD Assessment Test (CAT) score from baseline to 12 weeks after initiation of integrated therapy. Numerical values are shown in each panel. (A) FEV_1_, (B) FEV_1_/FVC ratio, (C) FVC and (D) CAT score. FEV_1_ increased from 2.27 to 2.73 L, FEV_1_/FVC from 82.5% to 86.9%, FVC from 2.75 to 3.14 L and CAT score decreased from 12 to 3.

After 12 weeks, spirometry improved: FVC increased to 3.14 L (104.6% predicted), FEV_1_ to 2.73 L (106.2% predicted; +0.46 L, +20.3%) and FEV_1_/FVC to 86.9%. The COPD Assessment Test (CAT) score decreased from 12 to 3 (Figure 2). Systemic inflammatory markers, including CRP, erythrocyte sedimentation rate, fibrinogen, platelet count, gamma‐globulin fraction and serum IgA, showed no clear overall improvement during the 12‐week follow‐up period (Table 1).

**TABLE 1:** Baseline and 12‐week clinical, respiratory, sleep, treatment, and inflammatory findings.

Parameter	Baseline	12 weeks	Notes
*Patient profile*			
Age (years)	40	41	
Sex	Female	Female	
Height (cm)	157.2	157.2	
Body weight (kg)	92	93	Essentially stable (+1 kg)
BMI (kg/m^2^)	37.2	37.6	Essentially stable
Smoking status	Current smoker	Current smoker	Continued smoking during follow‐up
Pack‐years	15	15	
Occupation/environmental exposure	Office worker; no occupational dust exposure; household secondhand smoke exposure during childhood/adolescence; active smoking from age 20	No change reported	
*Symptoms and patient‐reported outcomes*			
CAT score	12	3	
Epworth Sleepiness Scale	7	Not assessed	
*Respiratory function*			
FVC (L) (% predicted)	2.75 (91.6%)	3.14 (104.6%)	Baseline value was post‐bronchodilator
FEV_1_ (L) (% predicted)	2.27 (88.3%)	2.73 (106.2%)	+0.46 L, +20.3%; baseline value was post‐bronchodilator
FEV_1_/FVC (%)	82.5	86.9	+4.4 percentage points; baseline value was post‐bronchodilator
PEF (L/s)	5.17	4.82	
V50 (L/s)	3.98	4.50	
V25 (L/s)	0.97	1.82	
MMF (L/s)	Not available	3.75	
DLCO	Not measured	Not measured	Limitation
Bronchodilator response in FEV_1_ before treatment	2.08–2.27 L; +190 mL (+9.1%)	—	Negative by conventional criteria
Significant bronchodilator response	No	—	
*Asthma/eosinophilic phenotype markers*			
Wheeze on chest auscultation	Absent	Absent	No wheeze heard at any visit
Peripheral blood eosinophils (cells/μL)	96	95	Low
Total IgE (IU/mL)	11	Not repeated	Low
Sputum eosinophils	Negative	Not repeated	
Sputum cytology	Neutrophilic inflammation	Not repeated	
FeNO (ppb)	Not measured	Not measured	Limitation
*Systemic inflammatory markers*			
WBC (cells/μL)	9600	9500	
Neutrophils (%)	69	69	
CRP (mg/dL)	1.06	1.84	
ESR (mm/h)	48	44	Remained elevated
Fibrinogen (mg/dL)	458	544	
Platelet count (×10^9^/L)	539	481	Equivalent to 53.9 and 48.1 × 10^4^/μl
Gamma‐globulin (%)	20.7	19.8	
IgA (mg/dL)	443	435	
*Other laboratory data*			
Haemoglobin (g/dL)	10.9	11.8	
Albumin (g/dL)	3.9	4.2	
AST (U/L)	12	14	
ALT (U/L)	13	17	
Creatinine (mg/dL)	0.48	Not repeated	
eGFR (mL/min/1.73 m^2^)	111	Not repeated	
NT‐proBNP (pg/mL)	7	Not repeated	
*Sleep study and CPAP*			
AHI (events/h)	88.1	—	Baseline sleep study
ODI (events/h)	80.2	—	Baseline sleep study
Minimum SpO_2_ (%)	49	—	Baseline sleep study
Mean SpO_2_ (%)	89	—	Baseline sleep study
Time with SpO_2_ < 90%	178 min	—	Baseline sleep study
CPAP use (h/night)	—	7 h 50 min	Device‐recorded follow‐up data
CPAP days used (%)	—	100	Device‐recorded follow‐up data
Days with ≥ 4 h CPAP use (%)	—	100	Device‐recorded follow‐up data
Residual AHI on CPAP (events/h)	—	3	Device‐recorded follow‐up data
*Treatment and follow‐up*			
Inhaled therapy	Fluticasone furoate/umeclidinium/vilanterol initiated	Continued	100/62.5/25 micrograms once daily in the morning (Trelegy 100 Ellipta)
Adherence to inhaled therapy	Good	Good	By patient report
Inhaler technique	Not formally checked	Not formally checked	Limitation
Structured smoking‐cessation programme/pharmacotherapy	Not initiated	Not initiated	Patient remained a current smoker during follow‐up
Other medications	Antihypertensive therapy; headache medication for Chiari malformation type I	No additional medications during follow‐up	No new oral steroids, antibiotics, antiallergics, PPI, mucolytics, or other relevant medications
Adverse events	—	None	

*Note:* Baseline refers to assessment before initiation of inhaled fluticasone furoate/umeclidinium/vilanterol and continuous positive airway pressure. Baseline spirometric values used for diagnostic classification were post‐bronchodilator measurements. Pre‐treatment bronchodilator responsiveness showed an increase in FEV₁ from 2.08 to 2.27 L (Δ190 mL, +9.1%), which did not meet conventional criteria for a significant bronchodilator response. Sleep‐study parameters were obtained from baseline polysomnography, whereas CPAP adherence and residual AHI were obtained from device‐recorded follow‐up data. ‘Not repeated’ indicates that the test was not reassessed at 12 weeks.

Abbreviations: AHI, apnea‐hypopnea index; CAT, COPD Assessment Test; CPAP, continuous positive airway pressure; CRP, C‐reactive protein; DLCO, diffusing capacity of the lung for carbon monoxide; ESR, erythrocyte sedimentation rate; FeNO, fractional exhaled nitric oxide; FEV_1_, forced expiratory volume in 1 s; FVC, forced vital capacity; IgA, immunoglobulin A; IgE, immunoglobulin E; MMF, maximal mid‐expiratory flow; NT‐proBNP, N‐terminal pro‐B‐type natriuretic peptide; ODI, oxygen desaturation index; PEF, peak expiratory flow; SpO_2_, oxygen saturation; WBC, white blood cell count.

## 3 Discussion

This case illustrates the potential value of a treatable traits‐oriented approach in a symptomatic tobacco‐exposed patient with pre‐COPD and coexisting severe OSA. The improvement in symptoms and spirometry occurred despite continued smoking, essentially stable body weight, and no marked reduction in systemic inflammatory markers. Therefore, smoking cessation, weight loss, or resolution of systemic inflammation alone is unlikely to fully explain the observed improvement. The response should be interpreted as multifactorial, potentially involving inhaled therapy, effective CPAP treatment for severe OSA, improved respiratory management and natural variability.

The respiratory phenotype requires careful interpretation. Although improvement occurred after ICS‐containing triple therapy, several findings argued against a typical eosinophilic asthma phenotype: absence of wheeze during the clinical course, negative bronchodilator response by conventional criteria, low peripheral blood eosinophil count, low total IgE, negative sputum eosinophils and neutrophilic rather than eosinophilic sputum cytology. However, asthma or an asthma‐responsive component cannot be completely excluded because FeNO was not measured and because a single pre‐treatment reversibility assessment has limited sensitivity.

OSA was a clinically important comorbidity. Severe nocturnal hypoxaemia and sleep fragmentation could have contributed to dyspnoea, fatigue and poor patient‐reported outcomes. Effective CPAP treatment, documented by high adherence and low residual AHI, may therefore have contributed substantially to symptom improvement. This does not diminish the importance of tobacco exposure: smoking cessation remains the most important disease‐modifying intervention in pre‐COPD and should be prioritised even when other treatable traits are addressed.

This report has several limitations. It describes a single case and cannot establish causality or determine the relative contributions of inhaled therapy, CPAP, smoking‐related factors, or test variability. No structured smoking‐cessation intervention or pharmacotherapy was initiated despite continued smoking, which limits interpretation and reinforces that smoking cessation should remain central in pre‐COPD. Inhaler technique was not formally assessed, FeNO and DLCO were not measured and sputum cytology was not repeated at 12 weeks. The findings should therefore be considered hypothesis‐generating rather than definitive evidence for pharmacological treatment of pre‐COPD.

In conclusion, in a patient with pre‐COPD and severe OSA, integrated management with inhaled triple therapy and CPAP was associated with symptomatic and spirometric improvement over 12 weeks. The improvement was not accompanied by smoking cessation, substantial weight change, or clear improvement in systemic inflammatory markers. This case supports careful phenotyping and multifactorial management of selected patients with pre‐COPD, while underscoring the need for prospective studies.

## Author Contributions

**Norifumi Kudeken:** conceptualisation, methodology, investigation, writing – original draft, writing – review and editing.

## Funding

The author has nothing to report.

## Consent

The author declares that written informed consent was obtained for the publication of this manuscript and accompanying images using the consent form provided by the Journal.

## Conflicts of Interest

The author declares no conflicts of interest.

## Data Availability

The data that support the findings of this study are available on request from the corresponding author. The data are not publicly available due to privacy or ethical restrictions.
